# Two-Year Change in Blood Pressure Status and Left Ventricular Mass Index in Chinese Children

**DOI:** 10.3389/fmed.2021.708044

**Published:** 2021-08-24

**Authors:** Zilin Li, Yao Duan, Min Zhao, Costan G. Magnussen, Bo Xi

**Affiliations:** ^1^Department of Epidemiology, School of Public Health, Cheeloo College of Medicine, Shandong University, Jinan, China; ^2^Department of Nutrition and Food Hygiene, School of Public Health, Cheeloo College of Medicine, Shandong University, Jinan, China; ^3^Menzies Institute for Medical Research, University of Tasmania, Hobart, TAS, Australia; ^4^Research Centre of Applied and Preventive Cardiovascular Medicine, University of Turku, Turku, Finland; ^5^Centre for Population Health Research, Turku University Hospital, University of Turku, Turku, Finland

**Keywords:** children, blood pressure, hypertension, left ventricular mass index, change

## Abstract

**Background:** Elevated blood pressure (BP) is associated with target organ damage, such as left ventricular hypertrophy (LVH), in childhood. However, it is unclear if children who resolve elevated BP have reduced levels of left ventricular mass index (LVMI). This study aimed to examine the association between change in BP status over 2 years and LVMI among Chinese children.

**Methods:** Data were from 1,183 children aged 6–11 years at baseline in 2017 who were followed up in 2019 in the Huantai Childhood Cardiovascular Health Cohort Study. Change in BP status over 2 years from baseline to follow-up was categorized as: persistent normal BP, resolved elevated BP (elevated BP at baseline, normal BP at follow-up), incident elevated BP (normal BP at baseline, elevated BP at follow-up), and persistent elevated BP. Elevated BP status was defined according to national reference standards as systolic or diastolic BP levels ≥ sex-, age-, and height-specific 95th percentiles.

**Results:** LVMI levels were lowest in children with persistent normal BP (30.13 g/m^2.7^), higher in those with incident elevated BP (31.27 g/m^2.7^), and highest in those with persistent elevated BP (33.26 g/m^2.7^). However, LVMI levels in those who had resolved elevated BP (30.67 g/m^2.7^) were similar to those with persistent normal BP. In the fully adjusted model, compared with children with persistent normal BP, those with persistent elevated BP and incident elevated BP had higher LVMI at follow-up (ß = 3.131, *p* < 0.001; ß = 1.143, *p* = 0.041, respectively). In contrast, those who had resolved elevated BP did not have a significantly higher LVMI (ß = 0.545, *p* = 0.194) than those with persistent normal BP.

**Conclusion:** Developing or maintaining elevated BP over a 2-year period in childhood associated with higher levels of LVMI, but those able to resolve their elevated BP status over the same period had LVMI levels that were similar with those who had normal BP at both time points. Thus, it is important to identify children with elevated BP at early time and to take effective measures to lower their BP levels, thereby reducing high LVMI levels and related cardiovascular diseases in the future.

## Introduction

Left ventricular hypertrophy (LVH), a preclinical marker of cardiovascular damage, is an important risk factor for cardiovascular events and mortality (1). According to the American Academy of Pediatrics (AAP) guidelines on hypertension in children and adolescents, LVH is listed as the most common early target organ damage of pediatric hypertension (2). Our study showed that 32.4% had LVH among 333 children and adolescents (aged 6–17 years) who had hypertension based on blood pressure (BP) measurements on three separate visits (3). Clinical trials have demonstrated that antihypertensive therapy in children with hypertension can reduce the incidence of LVH, which indicates that maintaining a normal BP range in childhood has a positive effect on the prevention of LVH (4–6).

Left ventricular mass index (LVMI) is an important indicator for determining LVH. Several epidemiological studies have shown that pediatric elevated BP is positively associated with LVMI in childhood (7–9) or adulthood (10–12). However, these studies assessed BP status at only 1 survey point in childhood, and they did not consider how change in BP status during childhood might affect LVMI. The Beijing BP Cohort study has examined the association between change in BP status from childhood to adulthood and LVMI in adulthood, which showed that if children with elevated BP had normal BP in adulthood, the risk of high LVMI in adulthood was similar to those that had normal BP in both childhood and adulthood (13). However, it is unclear whether this finding is observed over a shorter time period in childhood. If this is to be shown, effective interventions that lower BP levels among children with elevated BP could be prioritized that might help reverse LVH to normal status in childhood as LVH in childhood usually presents as mild abnormal structure change.

Therefore, using data from the Huantai Children Cardiovascular Health Cohort Study, a prospective cohort study conducted from 2017 (baseline) to 2019 (follow-up), we aimed to examine the association between change in BP status over 2 years and LVMI among Chinese children. In addition, the hypothesis of this study was that during 2-year follow-up, children with normal BP at baseline but elevated BP at follow-up or with elevated BP at both periods would have higher LVMI levels, and in contrast, children who could resolve elevated BP over time would not have higher LVMI levels.

## Methods

### Study Design and Sample

**Figure 1:**
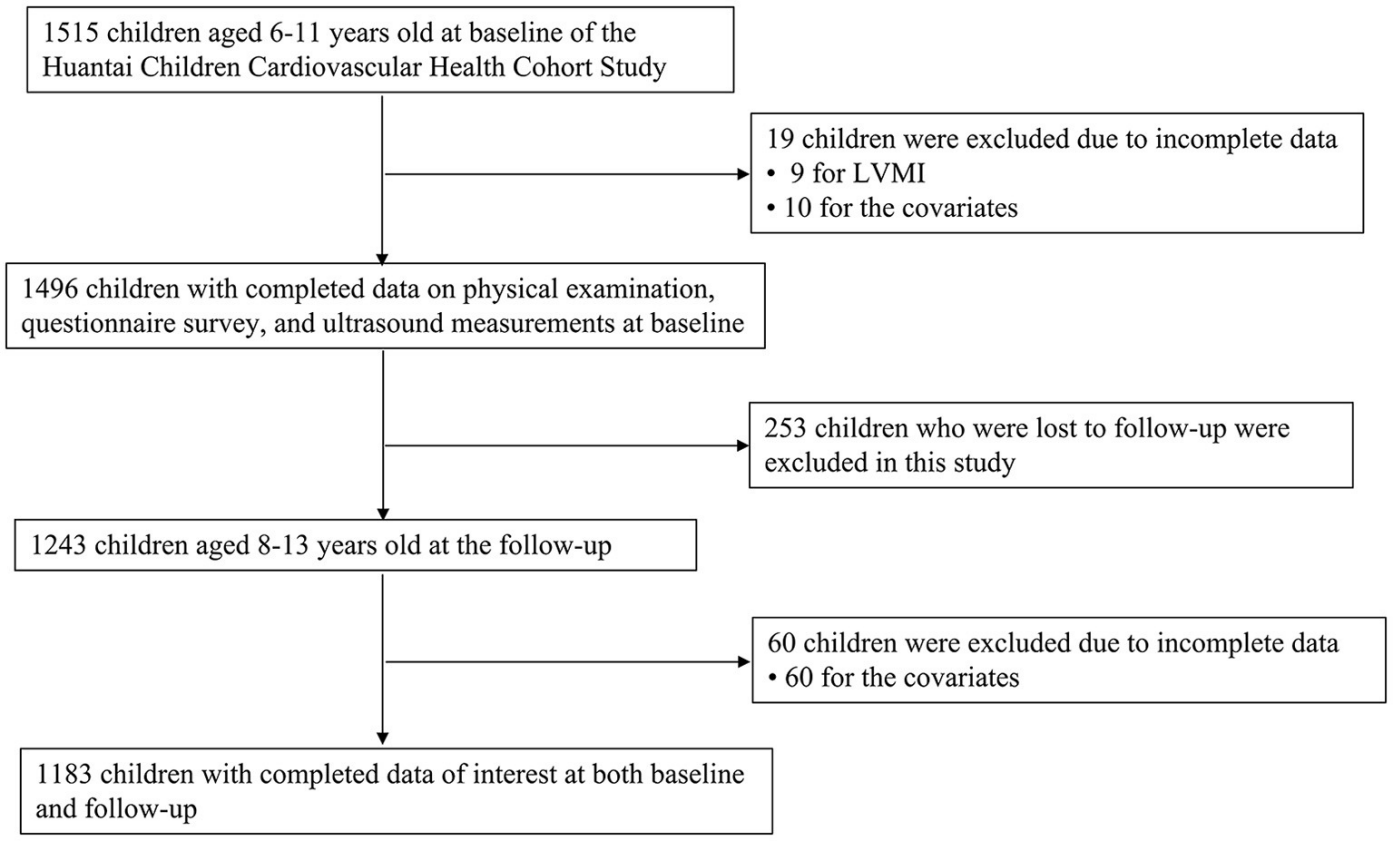
Flowchart of inclusion/exclusion of participants.

Data were from the baseline and follow-up of the Huantai Children Cardiovascular Health Cohort Study. At baseline, from November 2017 to January 2018, a total of 1,515 children aged 6–11 years from a public primary school in Huantai County, Zibo City, Shandong Province were selected using a convenient clustering sampling method. Detailed information on this cohort has been described elsewhere (14, 15). From November to December 2019, a total of 1,243 children who participated first at baseline were followed up. Participants were invited to complete a standardized questionnaire and undergo physical examinations and ultrasound measurements at both baseline and follow-up. After excluding participants who had missing information on the variables of interest at either baseline or follow-up (*n* = 332), a total of 1,183 children with complete data on age, sex, height, weight, systolic BP (SBP), diastolic BP (DBP), sleep duration, physical activity, intake of fruit and vegetables, intake of carbonated soft drink at both time points, and who had LVMI measured at follow-up, were included in the analysis sample. The flowchart of inclusion/exclusion of participants is shown as Figure 1. Written informed consent was obtained from participants and their parents/guardians. The study was approved by the Ethics Committee of the School of Public Health, Cheeloo College of Medicine, Shandong University.

### Physical Examination

Physical examinations were performed by trained staff based on a standardized protocol. Height was measured to the nearest 0.1 cm, and weight was measured to the nearest 0.1 kg using a standard scale (HGM 300, Universal Weight Electronics) with participants in light clothing without shoes. Height and weight were measured twice, and the average values were used for analysis. Body mass index (BMI, kg/m^2^) was calculated as the ratio of weight (in kg) over the square of height (in m). Overweight (including obesity) was defined as per the BMI classification reference in Chinese school-age children and adolescents as a BMI ≥ sex- and age-specific 85th percentile (16). BP was measured using electronic devices (OMRON HEM-7012 at baseline, OMRON HBP-1300 at follow-up). Both electronic devices have been validated and verified for use among Chinese youth (17, 18). In addition, we randomly selected 13 individuals to check the consistency between the two BP devices, and the intraclass correlation coefficients (ICC) for SBP [ICC = 0.81, 95% confidence interval (CI) = 0.42–0.94] and DBP (ICC = 0.76, 95% CI = 0.40–0.92) were relatively higher. After at least 10-min rest, BP was measured at the heart level of the right arm, in a seated position using the appropriate cuff size. BP was measured three times with the mean of the last two readings used for analysis based on the recommendations from several child BP Guidelines (2, 19). Information on age, sex, sleep duration, physical activity (<1 vs. ≥1 h/day) (20), intake of fruit and vegetables (<5 vs. ≥5 times/day) (21), and intake of carbonated soft drinks (<1 vs. ≥1 time/week) (22) was collected using a standardized questionnaire.

### Cardiac Assessment

Left ventricular structure was measured by a professional sonographer using Doppler ultrasound device (CX30, Philips, USA) with an S4-2 linear transducer of 2–4 MHz according to the 2010 Pediatric Cardiometry Guidelines of the American Society of Echocardiography (23). The sonographer was blinded to participant details at the time of measurement. Left ventricular internal diameter (LVID), interventricular septal thickness (IVST), and left ventricular posterior wall thickness (LVPWT) were measured. Left ventricular mass (LVM) was calculated using Devereux's formula: LVM (g) = 0.8 × [1.04 × (IVST + LVID + LVPWT)^3^ – (LVID)^3^] + 0.6 (24). LVMI (g/m^2.7^) was calculated as LVM (in g)/height^2.7^ (in m) to correct for body size (25).

### Definitions of Elevated BP and Change in BP Status

Elevated BP was defined as systolic and/or diastolic BP ≥ sex-, age-, and height-specific 95th percentile values according to the Chinese child BP references (26). Of note, there were no children who had taken hypertensive medication in this study. Based on BP status at baseline and follow-up, all participants were categorized into four groups: persistent normal BP (normal BP at both baseline and follow-up); resolved elevated BP (elevated BP at baseline but normal BP at follow-up); incident elevated BP (normal BP at baseline but elevated BP at follow-up); and persistent elevated BP (elevated BP at both baseline and follow-up). To test the stability of our results, we performed a sensitivity analysis by defining elevated BP as systolic and/or diastolic BP ≥ sex-, age-, and height-specific 95th percentile values according to the AAP guideline (2).

### Statistical Analysis

Categorical variables are presented as *n* (%) with differences between groups of the change in BP status compared using the chi-square test. Continuous variables are expressed as mean (standard deviation) with differences between groups of the change in BP status compared using analysis of variance. Covariance analysis was used to assess the difference in LVMI levels measured at follow-up across four groups of the change in BP status with adjustment for age, sex, intake of fruit and vegetables, intake of carbonated soft drink, physical activity, sleep duration, and weight status at baseline. Multivariable linear regression analysis was used to determine the association of change in BP status with LVMI at follow-up, with adjustment for the covariates mentioned above. We also performed subgroup analysis that stratified by weight status at baseline (overweight/obese vs. normal weight) to determine if weight status modified the observed association in the main analyses. In addition, we performed multivariate linear regression analyses to investigate association between elevated SBP/DBP at baseline or change in SBP/DBP (follow-up SBP/DBP values minus baseline SBP/DBP values, as a continuous variable) and LVMI at follow-up, after adjustment for potential covariates. A two-sided *p* < 0.05 was considered statistically significant. All analyses were performed using SAS 9.4 (SAS Institute, Cary, NC, US).

## Results

A total of 1,183 participants (52.5% boys) were included in this study. Mean value of LVMI was (30.31 ± 4.53) g/m^2.7^. Table 1 presents the characteristics of the study participants. The prevalence of elevated BP at baseline and follow-up were 14.8 and 11.8%, respectively. Children with persistent elevated BP had the highest prevalence of overweight and obesity at both baseline and follow-up, while those with persistent normal BP had the lowest prevalence.

**Table 1:** Characteristics of study participants at baseline and follow-up.

**Characteristics**	**All (*N* = 1,183)**	**Persistent normal BP (*N* = 997)**	**Resolved elevated BP (*N* = 98)**	**Incident elevated BP (*N* = 56)**	**Persistent elevated BP (*N* = 32)**	***p-*value**
**Baseline (2017)**						
Boys [*n* (%)]	621 (52.5)	521 (52.3)	49 (50.0)	35 (62.5)	16 (50.0)	0.458
Age [years, mean (SD)]	8.1 (1.5)	8.0 (1.5)	7.8 (1.5)	8.7 (1.4)	8.5 (1.8)	0.001
Height [cm, mean (SD)]	134.1 (10.4)	133.6 (10.4)	133.8 (8.8)	141.0 (9.7)	137.0 (11.0)	<0.001
Weight [kg, mean (SD)]	32.8 (9.5)	31.7 (8.9)	34.3 (7.8)	43.1 (11.8)	42.7 (12.7)	<0.001
BMI [kg/m^2^, mean (SD)]	17.9 (3.2)	17.5 (2.9)	19.0 (3.0)	21.4 (4.2)	22.2 (4.2)	<0.001
Overweight or obese [*n* (%)]	474 (40.1)	354 (35.5)	56 (57.1)	38 (67.9)	26 (81.3)	<0.001
SBP [mmHg, mean (SD)]	105.7 (9.1)	103.6 (7.6)	118.0 (7.5)	112.0 (5.6)	122.0 (7.1)	<0.001
DBP [mmHg, mean (SD)]	63.2 (6.5)	62.0 (5.8)	71.9 (6.1)	64.8 (5.1)	71.3 (5.6)	<0.001
Sleep duration [h/day, mean (SD)]	9.3 (0.5)	9.3 (0.5)	9.3 (0.4)	9.4 (0.5)	9.4 (0.4)	0.826
Physical activity [≥1 h/day, *n* (%)]	477 (40.3)	403 (40.4)	39 (39.8)	20 (35.7)	15 (46.7)	0.782
Fruit and vegetables [≥5 times/day, *n* (%)]	206 (17.4)	185 (18.6)	12 (12.2)	6 (10.7)	3 (9.4)	0.116
Carbonated soft drink [ <1 time/week, *n* (%)]	1,112 (94.0)	940 (94.3)	97 (99.0)	48 (85.7)	27 (84.4)	0.001
IVST [cm, mean (SD)]	0.59 (0.06)	0.58 (0.06)	0.59 (0.05)	0.64 (0.08)	0.64 (0.08)	<0.001
LVID [cm, mean (SD)]	3.9 (0.3)	3.9 (0.2)	4.0 (0.4)	4.1 (0.4)	4.1 (0.4)	<0.001
LVPWT [cm, mean (SD)]	0.60 (0.06)	0.60 (0.06)	0.60 (0.05)	0.66 (0.07)	0.65 (0.09)	<0.001
LVM [g, mean (SD)]	62.9 (16.2)	61.6 (14.9)	63.3 (15.5)	77.1(22.0)	77.0 (24.6)	<0.001
LVMI [g/m^2.7^, mean (SD)]	28.2 (4.4)	28.0 (4.1)	28.7 (5.3)	30.1(6.2)	32.1 (5.9)	<0.001
**Follow-up (2019)**						
Age [years, mean (SD)]	10.5 (1.5)	10.5 (1.5)	10.3 (1.5)	11.2 (1.5)	10.8 (1.7)	0.001
Height [cm, mean (SD)]	147.1 (11.2)	146.6 (11.3)	147.4 (9.6)	154.3 (10.5)	149.9 (11.3)	<0.001
Weight [kg, mean (SD)]	43.0 (12.8)	41.6 (12.0)	45.3 (10.6)	56.7 (15.4)	55.9 (16.1)	<0.001
BMI [kg/m^2^, mean (SD)]	19.5 (3.8)	19.0 (3.5)	20.7 (3.7)	23.5 (4.6)	24.4 (4.5)	<0.001
Overweight or obese [*n* (%)]	500 (42.3)	378 (37.9)	58 (59.2)	39 (69.6)	25 (78.1)	<0.001
SBP [mmHg, mean (SD)]	108.7 (10.0)	106.5 (8.3)	113.0 (6.3)	129.1 (6.8)	127.8 (7.1)	<0.001
DBP [mmHg, mean (SD)]	59.6 (6.7)	58.8 (6.3)	61.9 (6.8)	66.0 (7.1)	66.5 (8.0)	<0.001
Sleep duration [h/day, mean (SD)]	9.0 (0.7)	9.0 (0.7)	9.0 (0.7)	8.5 (0.8)	8.8 (0.7)	<0.001
Physical activity [≥1 h/day, *n* (%)]	677 (57.2)	558 (56.0)	60 (61.2)	37 (66.1)	22 (68.8)	0.186
Fruits and vegetables [≥5 times/day, *n* (%)]	300 (25.4)	254 (25.5)	18 (18.4)	18 (32.1)	10 (31.3)	0.214
Carbonated soft drink [ <1 time/week, *n* (%)]	1,095 (92.6)	920 (92.3)	92 (93.9)	52 (92.9)	31 (96.9)	0.745
IVST [cm, mean (SD)]	0.65 (0.04)	0.64 (0.04)	0.66 (0.04)	0.68 (0.05)	0.68 (0.06)	<0.001
LVID [cm, mean (SD)]	4.4 (0.3)	4.4 (0.3)	4.4 (0.3)	4.7 (0.4)	4.7 (0.4)	<0.001
LVPWT [cm, mean (SD)]	0.66 (0.05)	0.65 (0.04)	0.67 (0.05)	0.68 (0.04)	0.69 (0.06)	<0.001
LVM [g, mean (SD)]	85.9 (17.3)	84.3 (16.4)	88.6 (16.2)	101.5 (19.4)	100.8 (23.7)	<0.001
LVMI [g/m^2.7^, mean (SD)]	30.3 (4.5)	30.1 (4.5)	31.1 (4.8)	31.4 (4.5)	33.5 (4.7)	<0.001

*Chi-square test was performed to assess the differences of categorical variables between four BP status groups. Analysis of variance was performed to assess the differences of continuous variables between four BP status groups*.

*BMI, body mass index; BP, blood pressure; SBP, systolic blood pressure; DBP, diastolic blood pressure; IVST, interventricular septal thickness; LVID, left ventricular internal diameter; LVPWT, left ventricular posterior wall thickness; LVM, left ventricular mass; LVMI, left ventricular mass index; SD, standard deviation*.

Table 2 shows that after adjustment for sex and age at baseline, as well as intake of fruit and vegetables, intake of carbonated soft drink, physical activity, sleep duration, and weight status at baseline, LVMI at follow-up was highest in the persistent elevated BP group (33.26 g/m^2.7^), higher in the incident elevated BP group (31.27 g/m^2.7^), whereas LVMI was comparatively lower in the resolved elevated BP group (30.67 g/m^2.7^) and lowest in the persistent normal BP group (30.13 g/m^2.7^). Additionally, subgroup analyses based on weight status showed that when the analysis was limited to children with overweight/obesity, the results were similar to total sample, but this trend was not observed among children with normal weight. Results were consistent in sensitivity analysis that used the AAP guideline to define elevated BP (Supplementary Table 1).

**Table 2:** LVMI at follow-up across four groups of the change in BP status in the total sample and stratified by weight status.

**Change in BP status**	**LVMI at follow-up (g/m** ^****2.7****^ **)**	
	**Model 1**	**Model 2**
**Total (** ***N*** **=** **1,183)**		
Persistent normal BP (*n* = 997)	30.04 ± 0.13	30.13 ± 0.12
Resolved elevated BP (*n* = 98)	30.94 ± 0.40	30.67 ± 0.40
Incident elevated BP (*n* = 56)	31.89 ± 0.53	31.27 ± 0.54
Persistent elevated BP (*n* = 32)	33.98 ± 0.70	33.26 ± 0.71
*p*-value	<0.001	<0.001
**Normal weight (** ***N*** **=** **709)**		
Persistent normal BP (*n* = 643)	29.12 ± 0.14	29.12 ± 0.14
Resolved elevated BP (*n* = 42)	29.04 ± 0.54	29.03 ± 0.54
Incident elevated BP (*n* = 18)	29.54 ± 0.83	29.57 ± 0.83
Persistent elevated BP (*n* = 6)	29.92 ± 1.43	29.99 ± 1.44
*p*-value	0.901	0.878
**Overweight or obese (** ***N*** **=** **474)**		
Persistent normal BP (*n* = 354)	31.68 ± 0.22	31.66 ± 0.22
Resolved elevated BP (*n* = 56)	32.54 ± 0.57	32.48 ± 0.57
Incident elevated BP (*n* = 38)	33.10 ± 0.69	33.28 ± 0.69
Persistent elevated BP (*n* = 26)	34.98 ± 0.83	35.05 ± 0.84
*p*-value	<0.001	<0.001

*Data are presented as mean ± standard error. Covariance analysis was performed to assess the difference of LVMI between four BP status groups. Model 1: Adjusted for sex and age at baseline. Model 2: Model 1 covariates plus intake of fruit and vegetables, intake of carbonated soft drink, physical activity, sleep duration, and weight status (total sample only) at baseline*.

*LVMI, left ventricular mass index; BP, blood pressure*.

Compared with those in the persistent normal BP group, those in the persistent elevated BP group had a higher LVMI at follow-up (β = 3.131, *p* < 0.001) and those in the incident elevated BP group also had a higher LVMI (β = 1.143, *p* = 0.041) (Table 3). Conversely, those in the resolved elevated BP group did not have significantly higher LVMI compared with the persistent normal BP group (β = 0.545, *p* = 0.194). In subgroup analyses, we observed the same pattern in children with overweight/obesity at baseline but not for those who were normal weight at baseline. In addition, sensitivity analysis using the AAP guideline to define elevated BP showed similar results (Supplementary Table 2).

**Table 3:** Association of change in BP status with LVMI at follow-up in the total sample and stratified by weight status.

**Change in BP status**	**Model 1**				**Model 2**			
	**ß**	**SE**	**95% CI**	***p*-value**	**ß**	**SE**	**95% CI**	***p*-value**
**Total (** ***N*** **=** **1,183)**								
Persistent normal BP (*n* = 997)	Ref				Ref			
Resolved elevated BP (*n* = 98)	0.893	0.421	0.067–1.719	0.034	0.545	0.419	(−0.278)−1.368	0.194
Incident elevated BP (*n* = 56)	1.851	0.549	0.774–2.928	0.001	1.143	0.560	0.045–2.241	0.041
Persistent elevated BP (*n* = 32)	3.934	0.714	2.532–5.335	<0.001	3.131	0.722	1.715–4.547	<0.001
**Normal weight (** ***N*** **=** **709)**								
Persistent normal BP (*n* = 643)	Ref				Ref			
Resolved elevated BP (*n* = 42)	−0.082	0.558	(−1.178)−1.014	0.883	−0.095	0.561	(−1.196)−1.007	0.866
Incident elevated BP (*n* = 18)	0.417	0.841	(−1.234)−2.069	0.620	0.446	0.843	(−1.210)−2.101	0.597
Persistent elevated BP (*n* = 6)	0.800	1.437	(−2.022)−3.622	0.578	0.873	1.444	(−1.962)−3.709	0.546
**Overweight or obese (** ***N*** **=** **474)**								
Persistent normal BP (*n* = 354)	Ref				Ref			
Resolved elevated BP (*n* = 56)	0.867	0.609	(−0.329)−2.064	0.155	0.824	0.608	(−0.371)−2.018	0.176
Incident elevated BP (*n* = 38)	1.425	0.724	0.002–2.848	0.050	1.620	0.729	0.188–3.053	0.027
Persistent elevated BP (*n* = 26)	3.305	0.862	1.611–4.999	<0.001	3.394	0.868	1.689–5.099	<0.001

*LVMI, left ventricular mass index; BP, blood pressure; Ref, reference group; ß, linear regression coefficient; SE, standard error; 95% CI, 95% confidence interval*.

*Model 1: Adjusted for sex and age at baseline. Model 2: Model 1 covariates plus intake of fruit and vegetables, intake of carbonated soft drink, physical activity, sleep duration, and weight status (total sample only) at baseline*.

We further assessed which type of elevated BP (i.e., elevated SBP alone vs. elevated DBP alone) was more predictive for higher LVMI levels, and the results showed that children with elevated SBP alone had higher LVMI levels at follow-up (β = 1.437, *p* = 0.002). In contrast, there was no significant association between elevated DBP alone and LVMI levels at follow-up (β = 1.082, *p* = 0.156) (Supplementary Table 3). Additionally, per 1-mmHg change in SBP was positively associated with a 0.028-g/m^2.7^ change in LVMI at follow-up (*p* = 0.039), but there was no association between DBP change and LVMI at follow-up (β = 0.016, *p* = 0.682) (Supplementary Table 4).

## Discussion

Compared with children who had normal BP at both baseline and follow-up, children who developed incident elevated BP or had persistent elevated BP over the 2-year follow-up had higher levels of LVMI. Importantly, we found a substantial proportion of those with elevated BP levels at baseline were able to obtain normal levels 2 years later (98/130, ~75%) and that those able to resolve their elevated BP status in the time between baseline and follow-up had LVMI levels similar to those who had normal BP at both time points. These findings were most pronounced among children who were overweight or obese at baseline.

Previous studies have suggested a role of childhood elevated BP on LVMI both during childhood or adulthood. A meta-analysis of 10 studies, for example, showed a positive association between ambulatory systolic BP and LVMI in childhood, with a pooled correlation coefficient of 0.40 (95% CI = 0.30–0.50), and with those in the elevated BP group having a 6.53-g/m^2.7^ (95% CI = 4.73–8.33) higher LVMI, on average, than those in the normal BP group. However, as this meta-analysis was based mainly on children with kidney disease, when the analysis was restricted to healthy children, the association of ambulatory systolic BP with LVMI was slightly weakened but remained, with a pooled correlation coefficient of 0.32 (95% CI = 0.12–0.52) (27). Moreover, data from a longitudinal study of 1,061 adults aged 24–46 years with a mean follow-up of 28 years found that a 1-mmHg higher SBP level in childhood associated with a 0.08-g/m^2.7^ (95% CI = 0.01–0.14) higher LVMI in adulthood (11).

Nevertheless, our data are the first to assess the association between change in BP status in childhood and LVMI measured later in childhood. Our data accord with findings from child-to-adult cohorts. For example, data from the Beijing Blood Pressure Cohort that first measured BP among 1,259 children and adolescents aged 6–18 years at baseline who were followed up in adulthood 24 years later indicated that compared with those who had normal BP in childhood and adulthood, participants with elevated BP at both time points had increased odds of high LVMI in adulthood (OR = 2.2, 95% CI = 1.3–3.8). Conversely, for those with elevated BP in childhood but normal BP in adulthood, the odds of high LVMI was not significantly increased (OR = 1.3, 95% CI = 0.9–1.8) (13). As our findings are generally consistent with those from the Beijing Blood Pressure Cohort, collectively, they highlight the potential importance of maintaining normal BP levels throughout the early life-course. However, for those who are unable to avoid developing elevated BP in childhood, they are not destined to maintain the increase in associated risk if they are able to resolve their elevated BP status later in life.

In addition, our data suggest weight status as an important modifier of the association between change in BP status over 2 years and LVMI in Chinese children. Our recent study that used baseline data from this cohort showed that compared with children with both normal weight and normal BP, children with normal weight but elevated BP did not have higher odds of LVH (OR = 0.89, 95% CI = 0.30–2.62). In contrast, among children with overweight/obesity, those with normal BP (OR = 5.69, 95% CI = 3.39–9.55) and elevated BP (OR = 9.45, 95% CI = 5.47–16.33) (28) had higher odds of LVH. Research among 2,071 children aged 10–18 years indicated that a 1-unit increase in BMI and SBP was associated with a 0.37- and 0.08-g/m^2.7^, respectively, higher LVMI in childhood (29). While these findings suggest that BMI might have a stronger effect on LVMI compared with BP, they also suggest the two risk factors interact in their relation with LVMI/LVH. In our study, the association for BP and LVMI was much more pronounced among those with overweight or obesity. Our data suggest that those children who are overweight or obese might be most vulnerable to the effects of incident or persistent elevated BP on LVMI. As resolving excess weight status is difficult, even in childhood (30), the high proportion of children able to resolve elevated BP status and the potential benefit to LVMI, as we observed, suggest programs aimed at managing BP among overweight or obese children could be prioritized.

Furthermore, our study highlights that children with elevated SBP (vs. elevated DBP) should be prioritized for interventions. Previous study showed that isolated systolic hypertension is the most common form of hypertension in children (31). However, there is little evidence about the impact of isolated systolic hypertension in childhood on LVMI levels. Several studies indicated that isolated systolic hypertension in adulthood was positively associated with the higher risk of cardiovascular disease although isolated diastolic hypertension also played an important role (32, 33). Therefore, more studies are needed to investigate the relationship between different types of elevated BP during childhood and LVMI levels and cardiovascular disease in the future.

Finally, many studies have emphasized the importance of maintaining normal BP or lowering BP among participants with hypertension to reduce the burden of LVH, diabetes, and cardiovascular diseases (1, 4, 9, 34). A study conducted in 19 hypertensive children (median age of 15 years) showed that after 6-month ramipril (an antihypertensive medicine) treatment, the median ambulatory BP decreased by 11 and 7 mmHg for daytime SBP and DBP, respectively, and by 8 and 7 mmHg for nighttime SBP and DBP, respectively. Furthermore, the median LVMI levels decreased from 36.8 to 32.6 g/m^2.7^, and the prevalence of LVH decreased from 42 to 11% (4). Another study including 387 hypertensive adults with LVH indicated that after 2-year antihypertensive therapy, 242 (62.5%) subjects had regression of LVH, and LVM levels reduced by (49.8 ± 17.5) g in the LVH regression group. Additionally, compared with participants without LVH regression, those with LVH regression were at a reduced risk of cardiovascular disease (9). The third study presented that antihypertensive treatment cannot only reduce the risk of LVH and cardiovascular disease but also reduce the risk of type 2 diabetes (1). Thus, resolving elevated BP to normal status has a valuable clinical significance.

Several limitations of our study should be noted. First, because the selection of our sample was based on a convenient cluster sampling method in only one center in Huantai County, Zibo City, China, the generalizability of our findings is limited. Second, as participants were followed up for only 2 years, further studies with longer duration are needed to confirm our findings. Nevertheless, our data do accord with findings spanning childhood to adulthood. Third, the sample size of children in each subgroup was small, especially for the two later subgroups (incident elevated BP group: *n* = 56, persistent elevated BP: *n* = 32), and less when we further stratified by weight status, thus we were unable to show the meaningful results by different high BP stages due to low statistical power. In addition, the number of cases of LVH (≥95th percentile) was further lesser (*n* = 3 and *n* = 9 for the late two subgroups, respectively), which impeded us to investigate the association between change in BP status and LVH. Fourth, misclassification of the exposure was possible; for example, pediatric hypertension should be defined based on elevated BP on at least three occasions (2), but the definition of elevated BP we used was based on measurements from only one occasion at both time points. This might account, in-part, for the large proportion we observed who resolved their elevated BP status between time points. Moreover, our exposure does not capture individuals who might have had multiple, early, or late changes in BP status in the time between measurements. Reassuringly, the findings were consistent when we used an alternate definition of elevated BP status, though both these limitations with regard to misclassification remain. Thus, our findings might underestimate the observed relationships. Finally, although we have included a series of potential covariates for adjustment, other cardiovascular factors (such as salt consumption and other lifestyle factors) should be considered in the future.

In conclusion, we found that children with incident or persistent elevated BP over a 2-year interval had higher LVMI levels. However, children with elevated BP at baseline who were able to acquire a normal BP by follow-up had LVMI levels similar to those who had persistent normal BP at both time points. These data suggest that while maintaining a normal BP during childhood might be ideal to help prevent incident LVH, those with elevated BP in childhood may not be destined to maintain heightened risk if they are somehow able to obtain normal BP later in childhood.

## Data Availability Statement

The raw data supporting the conclusions of this article will be made available by the authors, without undue reservation.

## Ethics Statement

The studies involving human participants were reviewed and approved by Ethics Committee of the School of Public Health, Cheeloo College of Medicine, Shandong University. Written informed consent to participate in this study was provided by the participants' legal guardian/next of kin.

## Author Contributions

BX conceived and designed the study and will act as guarantor. MZ and CM provided comments and technical advice. ZL and YD analyzed the data and wrote the manuscript. All authors revised the manuscript critically for important intellectual content, gave final approval of the version to be published, and agree to be accountable for all aspects of the work.

## Conflict of Interest

The authors declare that the research was conducted in the absence of any commercial or financial relationships that could be construed as a potential conflict of interest.

## Publisher's Note

All claims expressed in this article are solely those of the authors and do not necessarily represent those of their affiliated organizations, or those of the publisher, the editors and the reviewers. Any product that may be evaluated in this article, or claim that may be made by its manufacturer, is not guaranteed or endorsed by the publisher.
