# Supplementary material for: Two-Year Change in Blood Pressure Status and Left Ventricular Mass Index in Chinese Children
Source: Front Med (Lausanne). 2021 Aug 24;8:708044. doi: 10.3389/fmed.2021.708044 (PMC8423149; doi:10.3389/fmed.2021.708044)
Supplement: Supplementary file 1 [file Data_Sheet_1.doc]

| **Supplementary Table 1. LVMI levels at follow-up across four groups of change in BP status in the total sample and stratified by weight status** | | |
| --- | --- | --- |
| **Change in BP status** | LVMI at follow-up, g/m^2.7^ | |
|  | Model 1 | Model 2 |
| **Total (N=1183)** |  |  |
| Persistent normal BP (n=926) | 29.96 ± 0.13 | 30.06 ± 0.13 |
| BP loss (n=121) | 30.58 ± 0.36 | 30.42 ± 0.36 |
| Incident elevated BP (n=79) | 31.87 ± 0.45 | 31.41 ± 0.45 |
| Persistent elevated BP (n=57) | 33.28 ± 0.53 | 32.65 ± 0.53 |
| *P* value | <0.001 | <0.001 |
| **Normal weight (N=709)** |  |  |
| Persistent normal BP (n=608) | 29.11 ± 0.14 | 29.12 ± 0.14 |
| BP loss (n=60) | 29.06 ± 0.46 | 29.02 ± 0.46 |
| Incident elevated BP (n=27) | 29.32 ± 0.68 | 29.31 ± 0.68 |
| Persistent elevated BP (n=14) | 29.93 ± 0.94 | 29.99 ± 0.94 |
| *P* value | 0.844 | 0.807 |
| **Overweight or obese (N=474)** |  |  |
| Persistent normal BP (n=318) | 31.53 ± 0.24 | 31.51 ± 0.23 |
| BP loss (n=61) | 32.15 ± 0.54 | 32.15 ± 0.54 |
| Incident elevated BP (n=52) | 33.33 ± 0.58 | 33.40 ± 0.58 |
| Persistent elevated BP (n=43) | 34.50 ± 0.64 | 34.50 ± 0.64 |
| *P* value | <0.001 | <0.001 |
| Data are presented as mean ± standard error. | | |
| Covariance analysis was performed to assess the difference of LVMI between four BP status groups. | | |
| Model 1: Adjusted for sex and age at baseline. | | |
| Model 2: Model 1 covariates plus intake of fruit and vegetables, intake of carbonated soft drink, physical activity, sleep duration, and weight status (total sample only) at baseline. | | |
| Abbreviations: LVMI, left ventricular mass index; BP, blood pressure. | | |
| *Elevated blood pressure status defined according to the guideline of the American Academy of Pediatrics. | | |

| **Supplementary Table 2. Association of change in BP status with LVMI at follow-up in the total sample and stratified by weight status** | | | | | | | | |
| --- | --- | --- | --- | --- | --- | --- | --- | --- |
| **Change in BP status** | Model 1 | | | | Model 2 | | | |
|  | *ß* | SE | 95%CI | *P* value | *ß* | SE | 95%CI | *P* value |
| **Total (N=1183)** |  |  |  |  |  |  |  |  |
| Persistent normal BP (n=926) | Ref |  |  |  | Ref |  |  | - |
| Resolved elevated BP (n=121) | 0.620 | 0.384 | (-0.134)-1.375 | 0.107 | 0.360 | 0.382 | (-0.390)-1.110 | 0.347 |
| Incident elevated BP (n=79) | 1.911 | 0.466 | 0.997-2.825 | <0.001 | 1.354 | 0.472 | 0.429-2.280 | 0.004 |
| Persistent elevated BP (n=57) | 3.322 | 0.543 | 2.256-4.387 | <0.001 | 2.588 | 0.553 | 1.504-3.673 | <0.001 |
| **Normal weight (N=709)** |  |  |  |  |  |  |  |  |
| Persistent normal BP (n=608) | Ref |  |  |  | Ref |  |  |  |
| Resolved elevated BP (n=60) | -0.052 | 0.479 | (-0.992)-0.887 | 0.913 | -0.099 | 0.481 | (-1.045)-0.846 | 0.837 |
| Incident elevated BP (n=27) | 0.201 | 0.690 | (-1.154)-1.555 | 0.771 | 0.193 | 0.692 | (-1.166)-1.552 | 0.780 |
| Persistent elevated BP (n=14) | 0.811 | 0.951 | (-1.056)-2.678 | 0.394 | 0.877 | 0.955 | (-0.998)-2.752 | 0.359 |
| **Overweight or obese (N=474)** |  |  |  |  |  |  |  |  |
| Persistent normal BP (n=318) | Ref |  |  |  | Ref |  |  |  |
| Resolved elevated BP (n=61) | 0.619 | 0.586 | (-0.533)-1.772 | 0.291 | 0.64 | 0.587 | (-0.513)-1.793 | 0.276 |
| Incident elevated BP (n=52) | 1.807 | 0.629 | 0.571-3.042 | 0.004 | 1.889 | 0.629 | 0.654-3.124 | 0.003 |
| Persistent elevated BP (n=43) | 2.973 | 0.684 | 1.629-4.317 | <0.001 | 2.983 | 0.686 | 1.635-4.331 | <0.001 |
| *ß*, linear regression coefficient; *SE*, standard error; 95%CI, 95% confidence interval. | | | | | | | | |
| Model 1: Adjusted for sex and age at baseline. | | | | | | | | |
| Model 2: Model 1 covariates plus intake of fruit and vegetables, intake of carbonated soft drink, physical activity, sleep duration, and weight status (total sample only) at baseline. | | | | | | | | |
| Abbreviations: LVMI, left ventricular mass index; BP, blood pressure; Ref, reference group. | | | | | | | | |
| Elevated blood pressure status defined according to the guideline of the American Academy of Pediatrics. | | | | | | | | |

| **Supplementary Table 3. Associations of elevated SBP and elevated DBP at baseline with LVMI levels at follow-up** | | | | | | | | |
| --- | --- | --- | --- | --- | --- | --- | --- | --- |
| **Change in BP status** | Model 1 | | | | Model 2 | | | |
|  | *ß* | SE | 95%CI | *P* value | *ß* | SE | 95%CI | *P* value |
| **Elevated SBP alone (n=84)** | 1.851 | 0.455 | 0.958- 2.743 | <0.001 | 1.437 | 0.450 | 0.554- 2.320 | 0.002 |
| **Elevated DBP alone (n=28)** | 1.504 | 0.777 | (-0.020)- 3.027 | 0.053 | 1.082 | 0.761 | (-0.412)- 2.576 | 0.156 |
| *ß*, linear regression coefficient; *SE*, standard error; 95%CI, 95% confidence interval. | | | | | | | | |
| Model 1: Adjusted for sex and age at baseline. | | | | | | | | |
| Model 2: Model 1 covariates plus intake of fruit and vegetables, intake of carbonated soft drink, physical activity, sleep duration, and weight status at baseline. | | | | | | | | |
| Abbreviations: LVMI, left ventricular mass index; SBP, systolic blood pressure; DBP, diastolic blood pressure; BP, blood pressure; Ref, reference group. | | | | | | | | |

| **Supplementary Table 4. Associations of SBP and DBP change during 2-year follow-up with LVMI level at follow-up** | | | | | | | | |
| --- | --- | --- | --- | --- | --- | --- | --- | --- |
|  | Model 1 | | | | Model 2 | | | |
|  | *ß* | SE | 95%CI | *P* value | *ß* | SE | 95%CI | *P* value |
| **SBP** | 0.041 | 0.013 | 0.015-0.068 | 0.002 | 0.028 | 0.013 | 0.001-0.054 | 0.039 |
| **DBP** | -0.005 | 0.017 | (-0.037)-0.028 | 0.769 | 0.007 | 0.016 | (-0.025)-0.039 | 0.682 |
| *ß*, linear regression coefficient; *SE*, standard error; 95%CI, 95% confidence interval. | | | | | | | | |
| Model 1: Adjusted for sex and age at baseline. | | | | | | | | |
| Model 2: Model 1 covariates plus intake of fruit and vegetables, intake of carbonated soft drink, physical activity, sleep duration, and weight status at baseline. | | | | | | | | |
| Abbreviations: LVMI, left ventricular mass index; SBP, systolic blood pressure; DBP, diastolic blood pressure. | | | | | | | | |
